# 

**Published:** 1855-01

**Authors:** 


### CONTENTS OF NO. II., VOL. II.

ORIGINAL COMMUNICATIONS.

Extract from Prof. Knowles's Introductory, . . . . 81
Case of Puerperal Fever—By P. Van Patten, M. D. . . . 91
Cases in Practice—By Prof. McGugin, . . . . . 97
Experiments with Indian Hemp—By Kirtley Ryland, M. D. 103

DEPARTMENT OF SELECTIONS.

Mental Derangement, its Symptoms and Treatment—By Roger G. Perkins, M. D. . . . . . . . 107
On the Treatment of Delirium Tremens—By A. Peddie, M.D. 117
Cases in the Massachusetts General Hospital—By R. M. Hodges, M. D. . . . . . . . . . . 124
Pumpkin Seed in Tenia, . . . . . . . . 128
Unsuccessful Treatment of Aæsthesia by Cold, . . . . 133
Prize Essay, . . . . . . . . . . 135
Iodine as a Topical Application, . . . . . . 136
Division of the Mucous Membrane to cure Fissures of the Rectum, . . . . . . . . . . 136
Diagnostic Sign of Choleraic Diarrhœa, . . . . . 136

EDITORIAL DEPARTMENT.

American Medical Profession, . . . . . . 137
Scalds by Steam—An Account of a Railroad Accident, . 140
Formula, . . . . . . . . . . . 144
Influence of Ether—A Suit against a Dental Surgeon, . . 145
Hydrocyanate of Iron in Epilepsy, . . . . . . 147
Medical Notes of Travel, . . . . . . . 148
Surgical Clinic, . . - . . . . . . . . 151
Present College Session, . . . . . . . . . 152
Testimonials of Gratitude, . . . . . . . . 154
Proceedings of the State Medical Society, . . . . 155

REVIEWS AND BIBLIOGRAPHICAL NOTICES.

Report of Poisoned Wounds—By A. F. Geeter, M. D. . 156
Simabra Cedron—By S. S. Purple, M. D. . . . . 156
Congestion of the Brain in Cholera—By I. M. Newman, M.D. 157
Necrosis of the Tibia, . . . . . . . . . 158
Nature, Signs, and Treatment of Childbed Fever—By Chas. D. Meigs, . . . . . . . . . 159
Healthy Skin—By Eras. Wilson, . . . . . . . 159
To our Exchanges, . . . . . . . . . 160
Surgical Instruments, . . . . . . . . . 160

